# Crystal structure of dimethyl *N*,*N*′-[(ethyne-1,2-di­yl)bis­(1,4-phenyl­enecarbon­yl)]bis­(l-alaninate)

**DOI:** 10.1107/S2056989019005826

**Published:** 2019-05-10

**Authors:** Frank Eissmann, Wilhelm Seichter, Edwin Weber

**Affiliations:** a TU Bergakademie Freiberg, Leipziger Str. 29, D-09596 Freiberg/Sachsen, Germany

**Keywords:** crystal structure, bis­(l-alaninate), mol­ecular tape formation, N—H⋯O=C and C—H⋯O hydrogen bonding

## Abstract

The di­phenyl­ethyne unit of the title mol­ecule, C_24_H_24_N_2_O_6_, deviates slightly from planarity. The l-alanine moieties adopt distorted helical conformations of opposite winding direction. Infinite ribbons of N—H⋯O=C-connected mol­ecules represent the basic supra­molecular entities of the crystal structure. These aggregates are linked by C—H⋯O hydrogen bonds involving the oxygen atoms of the methyl carboxyl­ate units. The crystal studied was refined as an inversion twin.

## Abstract 2

The title compound shows a mol­ecular framework with the di­phenyl­ethyne unit slightly deviating from planarity and the l-alanine moieties adopting a distorted helical conformation. The crystal structure features a two-dimensional network supported by N—H⋯O and C—H⋯O hydrogen bonding.

## Chemical context   

Currently, the design of solid porous framework materials has developed into a very significant aspect of supra­molecular crystal engineering (Desiraju *et al.*, 2011 ▸). In connection with it, mol­ecules frequently featuring a linear rigid structure and having coordinating or otherwise binding active functions as terminal groups are a desired structural unit in building such systems (Lin *et al.*, 2006 ▸; Hausdorf *et al.*, 2009 ▸; Zheng *et al.*, 2010 ▸). For this reason, the corresponding structural units are called ‘linker mol­ecules’. A particular type of linker mol­ecule consisting of a rod-like central unit and peptide terminal groups are promising in the assembly of bio-inspired framework materials including the subject chirality. Examples are the coordination polymers put together by *N*,*N*′-terephthalatoylbis(glycinate) (Eissmann *et al.*, 2010 ▸) and Cu^II^ (Kostakis *et al.*, 2005 ▸) or equivalent bis­(l-phenyl­alaninate) and Cu^II^ (Wisser *et al.*, 2008 ▸). In view of this applicability, the structural extension of this compound type is probably a future-oriented design. Precursor substances concerning this project have been prepared and structurally described in considerable numbers (Eissmann & Weber, 2011*a* ▸,*b* ▸). Here, we report for the first time the synthesis and crystal structure of a corres­ponding linker mol­ecule.
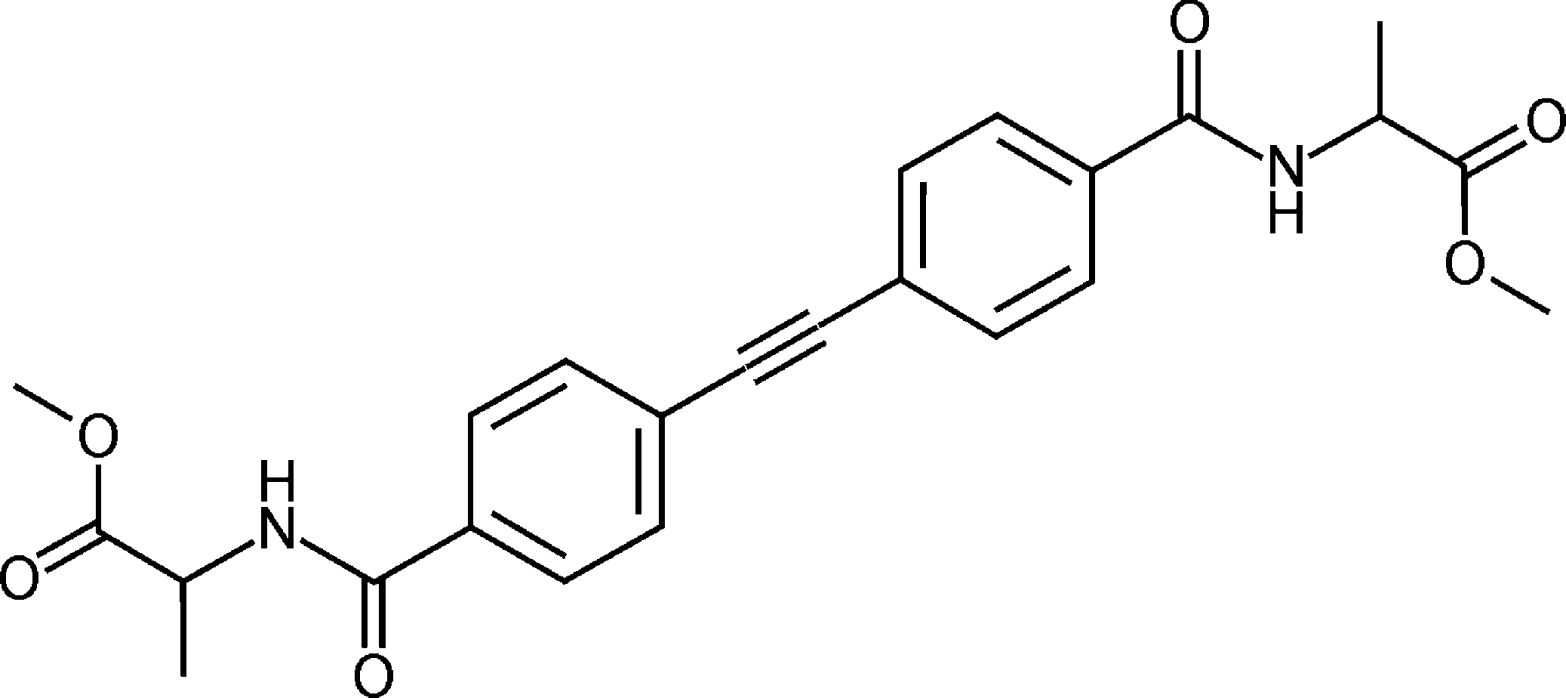


## Structural commentary   

**Figure 1:**
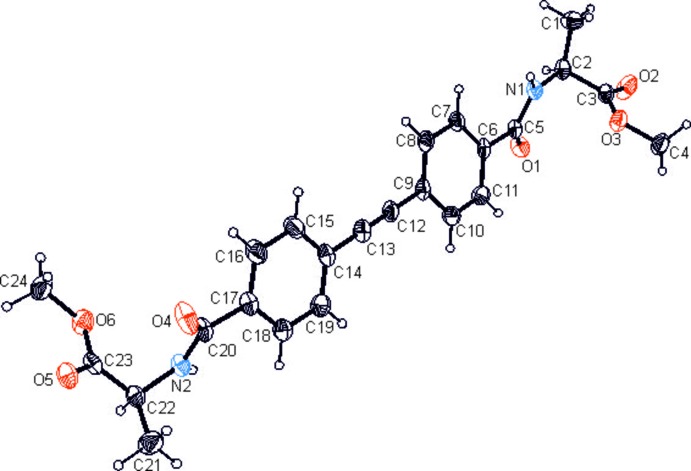
Perspective view of the mol­ecular structure of the title compound with the atom labeling. Displacement ellipsoids of non-H atoms are shown at the 50% probability level.

The title compound crystallizes in the monoclinic system (space group *P*2_1_) with one mol­ecule in the asymmetric unit. The mol­ecular structure (Fig. 1 ▸) is characterized by nearly planar *trans*-configured amide groups with ω_1_ = 169.9 (6)° and ω_2_ = 176.7 (6)°, which can be derived from torsion angles of −0.6 (5) and −3.3 (6)° for the atomic sequences C2—N1—C5—O1 and C22—N2—C20—O4. The least-squares planes through the amide groups are inclined at angles of 37.4 (9) and 40.1 (11)° with respect to the aromatic ring to which they are attached. The two l-alanine residues exist in distorted helical conformations of opposite winding direction with torsion angles φ_1_ = −70.2 (4)°, ψ_1_ = −19.4 (5)°, φ_2_ = 46.3 (5)° and ψ_2 =_ 49.4 (4)°. The central di­phenyl­ethyne element deviates slightly from planarity, showing a dihedral angle of 6.2 (2)° between the planes of the aromatic rings.

## Supra­molecular features   

**Figure 2:**
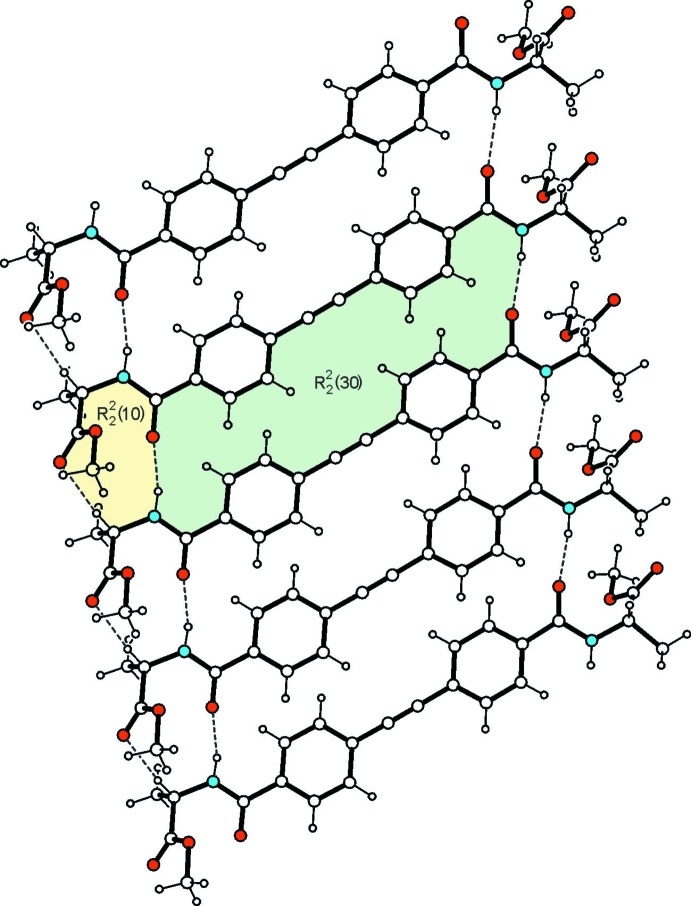
Structure of the mol­ecular ribbon including the mode of inter­molecular bonding in the crystal of the title compound. Dashed lines represent hydrogen bonds (Table 1 ▸). Ring motifs [graph sets 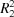
(30),
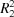
(10)] are marked by colour highlighting.

**Figure 3:**
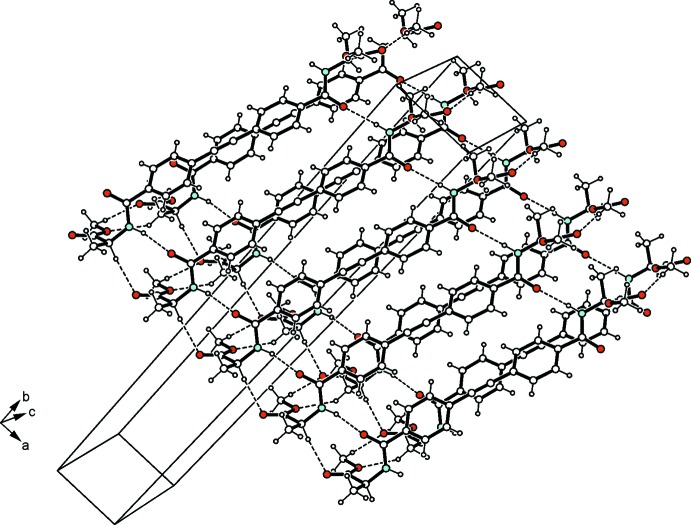
Packing diagram of the title compound. The inter­molecular contacts are shown as dashed lines.

In the crystal, each mol­ecule inter­acts with two neighbors *via* N—H⋯O=C_amide_ hydrogen bonding, thus generating infinite ribbons (Table 1 ▸, Fig. 2 ▸) which extend parallel to the *a* axis. These mol­ecular aggregates are additionally stabilized by a C—H⋯O bond (Desiraju & Steiner, 1999 ▸) between the ester oxygen atom O2 and the methine hydrogen of the stereogenic center C22. As shown in Fig. 2 ▸, within the tape structure the N—H⋯O bonds take part in two ring motifs that can be described by the graph sets 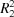
(30) and 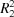
(10) (Etter *et al.*, 1990 ▸; Bernstein *et al.*, 1995 ▸). The ester groups participate to a different degree in mol­ecular association along the stacking direction (*c* axis) of the mol­ecular tapes. With the exception of O6, all ester oxygen atoms are involved in C—H⋯O inter­actions with meth­oxy hydrogen atoms acting as donors. The analysis of these inter­tape inter­actions reveals another two ring motifs of graph set 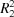
(8) and 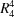
(26) (Fig. 3 ▸). According to the given pattern of hydrogen bonding, the crystal structure is composed of two-dimensional hydrogen-bonded layers connected by the linker mol­ecules in a zigzag pattern. The presence of the bulky headgroups prevents arene⋯arene inter­actions.

## Database survey   

A search in the Cambridge Structural Database (CSD, Version 5.38, update February 2017; Groom *et al*., 2016 ▸) revealed six hits for crystal structures of methyl *N*-benzoyl-l-alaninate and its para-substituted derivatives. Of particular inter­est are the structures of methyl *N*-(4-bromo­lbenzo­yl)-l-alaninate (IVOKIO; Eissmann & Weber, 2011*a* ▸) and methyl *N*-(4-ethynylbenzo­yl)-l-alaninate (PAHMIN; Eissmann & Weber, 2011*b* ▸). Their crystal packings are composed of structurally similar strands of N—H⋯O=C-bonded mol­ecules in which the amide N—H group acts as a donor and the amide O atom as an acceptor site. Unlike in the title compound, this inter­action is assisted by a C—H⋯O contact involving the l-alanine C_α_ methyl group as a donor and the *sp* ^3^-hybridized ester oxygen atom as an acceptor. In contrast, the crystal structure of methyl *N*-benzoyl-l-alaninate (XAZZON; Coghlan *et al*., 2000 ▸) is composed of zigzag strands of N—H⋯O=C-bonded mol­ecules. The ester group of the mol­ecule participates in inter­stand association via C—H⋯C=O-type hydrogen bonds, giving rise to two-dimensional supra­molecular networks.

## Synthesis and crystallization   

The title compound was prepared from methyl *N*-(4-bromo­benzo­yl)-l-alaninate (component-1) (Eissmann & Weber, 2011*a* ▸) and methyl *N*-(4-ethynylbenzo­yl)-l-alaninate (component-2) (Eissmann & Weber, 2011*b* ▸) *via* a Sonogashira–Hagihara cross-coupling reaction (Sonogashira *et al.* 1975 ▸) as follows. Component-1 (1.72 g, 6.0 mmol) and component-2 (1.39 g, 6.0 mmol) were dissolved in a degassed mixture of dry tri­methyl­amine (15 ml) and ethyl acetate (25 ml). To this solution, the catalyst being composed of tri­phenyl­phosphine (31.5 mg, 0.12 mmol), copper(I) iodide (22.9 mg, 0.12 mmol) and *trans*-di­chloro­bis­(tri­phenyl­phosphine)palladium(II) (42.1 mg, 0.06 mmol) was added. The mixture was stirred at room temperature away from light for 16 h. The precipitate which was formed was separated, washed three times with ethyl acetate (20 ml each) and suspended in an aqueous NH_4_Cl solution (100 ml). In this sequence, the isolated solid was washed with water (2 × 50 ml) and diethyl ether (4 × 25 ml). After drying in air, the product was obtained as a beige powder (1.39 g, 53%; m.p. 510–511 K; [α]_D_^20^ +61.4, 0.01 *M*, DMSO). ^1^H NMR (CDCl_3_): *δ*_H_ 1.42 (6H, *d*, ^3^ *J*_HH_ 7.30, CH—C*H*_3_), 3.66 (6H, *s*, O—C*H*_3_), 4.51 (2H, *qui*, ^3^ *J*_HH_ 7.15, C*H*), 7.71 (4H, *d*, ^3^ *J*_HH_ 8.35, Ar*H*), 7.96 (4H, *d*, ^3^ *J*_HH_ 8.40, Ar*H*), 8.93 (2H, *d*, ^3^ *J*_HH_ 6.90, N*H*). ^13^C NMR (DMSO-*d*_6_): *δ*_C_ 16.77 (CH*C*H_3_), 48.42 (*C*H), 51.99 (O*C*H_3_), 90.76 (*C*≡*C*), 124.95, 127.94, 131.49, 131.88, 133.76 (Ar*C*), 165.49 [Ar*C*(O)NH], 173.14 [*C*(O)OCH_3_]. IR (KBr): ν_max._ 3288 (NH), 1733 (C=O, ester), 1638 (C=O, amide), 1606, 1537 (Ar). MS (APCI): calculated for C_24_H_24_N_2_O_6_ (436.16), found 435.1 [*M* − H]^−^. Analysis calculated for C_24_H_24_N_2_O_6_: C, 66.04; H, 5.54; N, 6.42; found: C, 66.23; H, 5.58; N, 6.45%. Colorless crystals suitable for X-ray diffraction were obtained from a solution of DMSO upon slow evaporation of the solvent at room temperature.

## Refinement   

Crystal data, data collection and structure refinement details are summarized in Table 2 ▸. The hydrogen atoms were positioned geometrically and refined isotropically using a riding model with C—H = 0.98 Å and *U*_iso_(H) = 1.5*U*_eq_(C) for methyl and C—H = 0.95 Å and *U*_iso_(H) = 1.2*U*_eq_(C) for aryl H atoms. The crystal studied was refined as an inversion twin.

## Supplementary Material

Crystal structure: contains datablock(s) I. DOI: 10.1107/S2056989019005826/zp2034sup1.cif

Structure factors: contains datablock(s) I. DOI: 10.1107/S2056989019005826/zp2034Isup2.hkl

Supporting information file. DOI: 10.1107/S2056989019005826/zp2034Isup3.cml

CCDC reference: 1912918

Additional supporting information:  crystallographic information; 3D view; checkCIF report

## Figures and Tables

**Table 1:** Hydrogen-bond geometry (Å, °)

*D*—H⋯*A*	*D*—H	H⋯*A*	*D*⋯*A*	*D*—H⋯*A*
N1—H1⋯O1^i^	0.89 (1)	2.11 (2)	2.982 (4)	168 (3)
N2—H2⋯O4^ii^	0.89 (1)	1.93 (2)	2.799 (4)	165 (5)
C1—H1*C*⋯O2^i^	0.98	2.58	3.532 (6)	164
C4—H4*B*⋯O2^iii^	0.98	2.36	3.340 (5)	176
C21—H21*B*⋯O6^iii^	0.98	2.53	3.315 (5)	137
C22—H22⋯O5^ii^	1.00	2.38	3.380 (5)	174
C24—H24*B*⋯O5^iv^	0.98	2.46	3.394 (5)	158

Symmetry codes: (i) 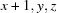; (ii) 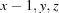; (iii) 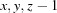; (iv) 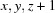.

**Table 2:** Experimental details

Crystal data	
Chemical formula	C_24_H_24_N_2_O_6_
*M*_r_	436.45
Crystal system, space group	Monoclinic, *P*2_1_
Temperature (K)	153
*a*, *b*, *c* (Å)	4.9409 (4), 39.015 (3), 5.8447 (4)
β (°)	100.905 (3)
*V* (Å^3^)	1106.34 (14)
*Z*	2
Radiation type	Mo *K*α
μ (mm^−1^)	0.10
Crystal size (mm)	0.25 × 0.18 × 0.13

Data collection	
Diffractometer	Bruker APEXII CCD area-detector
Absorption correction	Multi-scan (*SADABS*; Bruker, 2008 ▸)
*T*_min_, *T*_max_	0.977, 0.988
No. of measured, independent and observed [*I* > 2σ(*I*)] reflections	10506, 5192, 3859
*R*_int_	0.034
(sin θ/λ)_max_ (Å^−1^)	0.672

Refinement	
*R*[*F* ^2^ > 2σ(*F* ^2^)], *wR*(*F* ^2^), *S*	0.055, 0.125, 1.00
No. of reflections	5192
No. of parameters	302
No. of restraints	3
H-atom treatment	H atoms treated by a mixture of independent and constrained refinement
Δρ_max_, Δρ_min_ (e Å^−3^)	0.16, −0.23
Absolute structure	Refined as an inversion twin

Computer programs: *APEX2* and, *SAINT* (Bruker, 2014 ▸), *SHELXS* (Sheldrick, 2015*a* ▸), *SHELXL2014* (Sheldrick, 2015*b* ▸), *ORTEP-3 for Windows* (Farrugia, 2012 ▸) and *SHELXTL* (Sheldrick, 2008 ▸).

## supplementary crystallographic information

### Crystal data

**Table:**

C_24_H_24_N_2_O_6_	*F*(000) = 460
*M**_r_* = 436.45	*D*_x_ = 1.310 Mg m^−^^3^
Monoclinic, *P*2_1_	Mo *K*α radiation, λ = 0.71073 Å
*a* = 4.9409 (4) Å	Cell parameters from 2881 reflections
*b* = 39.015 (3) Å	θ = 3.6–26.5°
*c* = 5.8447 (4) Å	µ = 0.10 mm^−^^1^
β = 100.905 (3)°	*T* = 153 K
*V* = 1106.34 (14) Å^3^	Irregular, colourless
*Z* = 2	0.25 × 0.18 × 0.13 mm

### Data collection

**Table:**

Bruker APEXII CCD area-detector diffractometer	3859 reflections with *I* > 2σ(*I*)
Radiation source: sealed x-ray tube	*R*_int_ = 0.034
φ and ω scans	θ_max_ = 28.5°, θ_min_ = 2.1°
Absorption correction: multi-scan (SADABS; Bruker, 2008)	*h* = −4→6
*T*_min_ = 0.977, *T*_max_ = 0.988	*k* = −52→49
10506 measured reflections	*l* = −7→7
5192 independent reflections	

### Refinement

**Table:**

Refinement on *F*^2^	Hydrogen site location: mixed
Least-squares matrix: full	H atoms treated by a mixture of independent and constrained refinement
*R*[*F*^2^ > 2σ(*F*^2^)] = 0.055	*w* = 1/[σ^2^(*F*_o_^2^) + (0.0519*P*)^2^ + 0.3338*P*] where *P* = (*F*_o_^2^ + 2*F*_c_^2^)/3
*wR*(*F*^2^) = 0.125	(Δ/σ)_max_ < 0.001
*S* = 1.00	Δρ_max_ = 0.16 e Å^−^^3^
5192 reflections	Δρ_min_ = −0.23 e Å^−^^3^
302 parameters	Absolute structure: Refined as an inversion twin
3 restraints	

### Special details

**Table:**

Geometry. All esds (except the esd in the dihedral angle between two l.s. planes) are estimated using the full covariance matrix. The cell esds are taken into account individually in the estimation of esds in distances, angles and torsion angles; correlations between esds in cell parameters are only used when they are defined by crystal symmetry. An approximate (isotropic) treatment of cell esds is used for estimating esds involving l.s. planes.
Refinement. Refined as a 2-component inversion twin.

### Fractional atomic coordinates and isotropic or equivalent isotropic displacement parameters (Å^2^)

**Table:**

	*x*	*y*	*z*	*U*_iso_*/*U*_eq_	
O1	−0.3228 (6)	0.27103 (7)	1.3737 (5)	0.0359 (7)	
O2	−0.1565 (8)	0.36026 (8)	1.6451 (5)	0.0492 (9)	
O3	0.0032 (6)	0.34262 (6)	1.3334 (5)	0.0351 (7)	
O4	0.6614 (6)	0.01503 (7)	0.0365 (6)	0.0438 (8)	
O5	0.5951 (6)	−0.06167 (7)	−0.1874 (5)	0.0332 (6)	
O6	0.4374 (6)	−0.05575 (7)	0.1439 (5)	0.0372 (7)	
N1	0.1280 (7)	0.27972 (8)	1.5074 (6)	0.0262 (7)	
H1	0.301 (4)	0.2773 (9)	1.489 (6)	0.016 (9)*	
N2	0.2024 (7)	0.00595 (8)	−0.0317 (6)	0.0275 (7)	
H2	0.039 (5)	0.0127 (12)	−0.002 (8)	0.044 (13)*	
C1	0.3251 (9)	0.31396 (11)	1.8526 (7)	0.0395 (10)	
H1A	0.3866	0.2931	1.9401	0.059*	
H1B	0.2791	0.3314	1.9594	0.059*	
H1C	0.4728	0.3225	1.7771	0.059*	
C2	0.0727 (8)	0.30608 (9)	1.6691 (7)	0.0279 (8)	
H2A	−0.0708	0.2968	1.7520	0.034*	
C3	−0.0422 (8)	0.33900 (9)	1.5482 (7)	0.0283 (8)	
C4	−0.1041 (10)	0.37401 (11)	1.2158 (7)	0.0420 (11)	
H4A	−0.2869	0.3789	1.2501	0.063*	
H4B	−0.1182	0.3713	1.0473	0.063*	
H4C	0.0206	0.3931	1.2708	0.063*	
C5	−0.0835 (8)	0.26402 (9)	1.3680 (7)	0.0251 (8)	
C6	−0.0077 (8)	0.23641 (9)	1.2131 (7)	0.0272 (9)	
C7	0.2173 (9)	0.21521 (9)	1.2828 (7)	0.0299 (9)	
H7	0.3311	0.2183	1.4316	0.036*	
C8	0.2781 (8)	0.18931 (10)	1.1366 (7)	0.0312 (9)	
H8	0.4310	0.1746	1.1878	0.037*	
C9	0.1184 (8)	0.18472 (9)	0.9175 (7)	0.0293 (8)	
C10	−0.1098 (9)	0.20622 (10)	0.8463 (8)	0.0356 (10)	
H10	−0.2211	0.2035	0.6960	0.043*	
C11	−0.1735 (9)	0.23150 (10)	0.9951 (7)	0.0320 (9)	
H11	−0.3313	0.2455	0.9477	0.038*	
C12	0.1832 (9)	0.15849 (9)	0.7647 (7)	0.0309 (9)	
C13	0.2348 (9)	0.13652 (10)	0.6362 (7)	0.0327 (9)	
C14	0.2895 (9)	0.10953 (10)	0.4845 (7)	0.0308 (9)	
C15	0.5208 (9)	0.08859 (11)	0.5503 (8)	0.0402 (11)	
H15	0.6465	0.0928	0.6918	0.048*	
C16	0.5651 (9)	0.06161 (11)	0.4073 (8)	0.0414 (11)	
H16	0.7246	0.0477	0.4506	0.050*	
C17	0.3828 (8)	0.05450 (9)	0.2040 (7)	0.0275 (8)	
C18	0.1575 (9)	0.07580 (10)	0.1360 (8)	0.0353 (10)	
H18	0.0335	0.0716	−0.0065	0.042*	
C19	0.1123 (9)	0.10325 (10)	0.2755 (8)	0.0366 (10)	
H19	−0.0417	0.1179	0.2269	0.044*	
C20	0.4274 (8)	0.02379 (9)	0.0616 (7)	0.0287 (9)	
C21	0.2291 (10)	−0.01405 (12)	−0.4265 (7)	0.0432 (11)	
H21A	0.0730	0.0008	−0.4902	0.065*	
H21B	0.2231	−0.0348	−0.5222	0.065*	
H21C	0.4019	−0.0018	−0.4272	0.065*	
C22	0.2127 (8)	−0.02395 (9)	−0.1792 (6)	0.0266 (8)	
H22	0.0349	−0.0365	−0.1866	0.032*	
C23	0.4398 (8)	−0.04847 (9)	−0.0795 (6)	0.0260 (8)	
C24	0.6460 (10)	−0.07959 (11)	0.2541 (7)	0.0441 (11)	
H24A	0.6093	−0.1023	0.1831	0.066*	
H24B	0.6422	−0.0810	0.4209	0.066*	
H24C	0.8278	−0.0716	0.2330	0.066*	

### Atomic displacement parameters (Å^2^)

**Table:**

	*U*^11^	*U*^22^	*U*^33^	*U*^12^	*U*^13^	*U*^23^
O1	0.0247 (15)	0.0326 (15)	0.0523 (18)	0.0022 (12)	0.0120 (13)	−0.0084 (13)
O2	0.077 (2)	0.0366 (17)	0.0371 (17)	0.0282 (16)	0.0196 (17)	0.0017 (13)
O3	0.0475 (18)	0.0253 (14)	0.0347 (16)	0.0060 (13)	0.0135 (13)	0.0029 (12)
O4	0.0220 (16)	0.0335 (16)	0.080 (2)	−0.0023 (12)	0.0185 (15)	−0.0213 (15)
O5	0.0339 (16)	0.0292 (14)	0.0390 (15)	0.0031 (12)	0.0135 (12)	−0.0066 (12)
O6	0.0442 (18)	0.0350 (16)	0.0353 (16)	0.0093 (13)	0.0149 (13)	0.0002 (12)
N1	0.0209 (17)	0.0217 (16)	0.0377 (18)	0.0026 (13)	0.0095 (14)	−0.0032 (13)
N2	0.0225 (17)	0.0262 (16)	0.0347 (18)	0.0016 (14)	0.0077 (14)	−0.0056 (14)
C1	0.035 (2)	0.042 (2)	0.039 (2)	0.011 (2)	−0.0006 (19)	−0.0103 (19)
C2	0.030 (2)	0.0208 (17)	0.036 (2)	0.0049 (16)	0.0149 (17)	−0.0025 (16)
C3	0.031 (2)	0.0214 (18)	0.033 (2)	0.0027 (16)	0.0076 (17)	−0.0020 (16)
C4	0.063 (3)	0.027 (2)	0.035 (2)	0.008 (2)	0.008 (2)	0.0057 (18)
C5	0.025 (2)	0.0184 (16)	0.033 (2)	−0.0008 (15)	0.0086 (16)	−0.0001 (15)
C6	0.030 (2)	0.0154 (17)	0.037 (2)	0.0006 (15)	0.0098 (18)	0.0013 (15)
C7	0.032 (2)	0.0203 (19)	0.036 (2)	0.0031 (16)	0.0037 (17)	−0.0041 (16)
C8	0.034 (2)	0.0200 (18)	0.039 (2)	0.0081 (17)	0.0074 (17)	−0.0035 (17)
C9	0.038 (2)	0.0143 (17)	0.037 (2)	0.0024 (16)	0.0117 (17)	0.0010 (15)
C10	0.038 (2)	0.030 (2)	0.037 (2)	0.0063 (18)	0.0044 (18)	−0.0042 (17)
C11	0.030 (2)	0.028 (2)	0.037 (2)	0.0093 (18)	0.0031 (18)	−0.0013 (17)
C12	0.039 (2)	0.0193 (18)	0.034 (2)	0.0031 (16)	0.0066 (17)	−0.0016 (16)
C13	0.037 (2)	0.0241 (19)	0.039 (2)	0.0021 (18)	0.0112 (18)	−0.0001 (18)
C14	0.036 (2)	0.0209 (18)	0.037 (2)	−0.0002 (16)	0.0104 (18)	−0.0053 (16)
C15	0.039 (3)	0.032 (2)	0.046 (3)	0.0033 (19)	−0.003 (2)	−0.0124 (19)
C16	0.031 (2)	0.033 (2)	0.056 (3)	0.0105 (19)	−0.002 (2)	−0.015 (2)
C17	0.024 (2)	0.0231 (18)	0.038 (2)	−0.0027 (15)	0.0127 (17)	−0.0030 (16)
C18	0.040 (3)	0.028 (2)	0.037 (2)	0.0070 (18)	0.0044 (19)	−0.0044 (17)
C19	0.038 (3)	0.029 (2)	0.042 (2)	0.0114 (18)	0.005 (2)	−0.0021 (18)
C20	0.023 (2)	0.0228 (18)	0.042 (2)	−0.0019 (16)	0.0107 (17)	−0.0037 (16)
C21	0.053 (3)	0.047 (3)	0.030 (2)	0.016 (2)	0.009 (2)	0.0023 (19)
C22	0.026 (2)	0.0246 (19)	0.030 (2)	0.0004 (15)	0.0079 (16)	−0.0058 (15)
C23	0.028 (2)	0.0220 (18)	0.0293 (19)	−0.0055 (16)	0.0091 (16)	−0.0061 (15)
C24	0.056 (3)	0.037 (2)	0.037 (2)	0.018 (2)	0.003 (2)	0.001 (2)

### Geometric parameters (Å, º)

**Table:**

O1—C5	1.220 (5)	C8—H8	0.9500
O2—C3	1.203 (5)	C9—C10	1.404 (6)
O3—C3	1.324 (5)	C9—C12	1.433 (5)
O3—C4	1.455 (5)	C10—C11	1.390 (5)
O4—C20	1.241 (5)	C10—H10	0.9500
O5—C23	1.198 (4)	C11—H11	0.9500
O6—C23	1.338 (4)	C12—C13	1.198 (5)
O6—C24	1.446 (5)	C13—C14	1.435 (5)
N1—C5	1.345 (5)	C14—C19	1.384 (6)
N1—C2	1.457 (4)	C14—C15	1.398 (6)
N1—H1	0.886 (14)	C15—C16	1.387 (6)
N2—C20	1.337 (5)	C15—H15	0.9500
N2—C22	1.457 (5)	C16—C17	1.377 (6)
N2—H2	0.894 (14)	C16—H16	0.9500
C1—C2	1.514 (6)	C17—C18	1.386 (6)
C1—H1A	0.9800	C17—C20	1.499 (5)
C1—H1B	0.9800	C18—C19	1.390 (6)
C1—H1C	0.9800	C18—H18	0.9500
C2—C3	1.523 (5)	C19—H19	0.9500
C2—H2A	1.0000	C21—C22	1.513 (6)
C4—H4A	0.9800	C21—H21A	0.9800
C4—H4B	0.9800	C21—H21B	0.9800
C4—H4C	0.9800	C21—H21C	0.9800
C5—C6	1.499 (5)	C22—C23	1.505 (5)
C6—C7	1.384 (5)	C22—H22	1.0000
C6—C11	1.392 (6)	C24—H24A	0.9800
C7—C8	1.393 (5)	C24—H24B	0.9800
C7—H7	0.9500	C24—H24C	0.9800
C8—C9	1.383 (6)		
			
C3—O3—C4	115.2 (3)	C10—C11—C6	120.5 (4)
C23—O6—C24	115.7 (3)	C10—C11—H11	119.8
C5—N1—C2	119.7 (3)	C6—C11—H11	119.8
C5—N1—H1	122 (2)	C13—C12—C9	179.4 (5)
C2—N1—H1	117 (2)	C12—C13—C14	178.1 (4)
C20—N2—C22	122.6 (3)	C19—C14—C15	119.2 (4)
C20—N2—H2	119 (3)	C19—C14—C13	120.9 (4)
C22—N2—H2	119 (3)	C15—C14—C13	119.9 (4)
C2—C1—H1A	109.5	C16—C15—C14	119.4 (4)
C2—C1—H1B	109.5	C16—C15—H15	120.3
H1A—C1—H1B	109.5	C14—C15—H15	120.3
C2—C1—H1C	109.5	C17—C16—C15	121.4 (4)
H1A—C1—H1C	109.5	C17—C16—H16	119.3
H1B—C1—H1C	109.5	C15—C16—H16	119.3
N1—C2—C1	111.9 (3)	C16—C17—C18	119.1 (4)
N1—C2—C3	113.2 (3)	C16—C17—C20	120.0 (4)
C1—C2—C3	110.0 (3)	C18—C17—C20	121.0 (4)
N1—C2—H2A	107.2	C17—C18—C19	120.2 (4)
C1—C2—H2A	107.2	C17—C18—H18	119.9
C3—C2—H2A	107.2	C19—C18—H18	119.9
O2—C3—O3	123.5 (4)	C14—C19—C18	120.6 (4)
O2—C3—C2	122.0 (3)	C14—C19—H19	119.7
O3—C3—C2	114.5 (3)	C18—C19—H19	119.7
O3—C4—H4A	109.5	O4—C20—N2	122.0 (3)
O3—C4—H4B	109.5	O4—C20—C17	121.6 (3)
H4A—C4—H4B	109.5	N2—C20—C17	116.4 (3)
O3—C4—H4C	109.5	C22—C21—H21A	109.5
H4A—C4—H4C	109.5	C22—C21—H21B	109.5
H4B—C4—H4C	109.5	H21A—C21—H21B	109.5
O1—C5—N1	121.8 (3)	C22—C21—H21C	109.5
O1—C5—C6	122.1 (4)	H21A—C21—H21C	109.5
N1—C5—C6	116.0 (3)	H21B—C21—H21C	109.5
C7—C6—C11	119.2 (3)	N2—C22—C23	112.7 (3)
C7—C6—C5	122.0 (4)	N2—C22—C21	112.0 (3)
C11—C6—C5	118.7 (3)	C23—C22—C21	111.2 (3)
C6—C7—C8	120.5 (4)	N2—C22—H22	106.8
C6—C7—H7	119.7	C23—C22—H22	106.8
C8—C7—H7	119.7	C21—C22—H22	106.8
C9—C8—C7	120.7 (4)	O5—C23—O6	123.2 (4)
C9—C8—H8	119.6	O5—C23—C22	125.0 (3)
C7—C8—H8	119.6	O6—C23—C22	111.6 (3)
C8—C9—C10	118.9 (4)	O6—C24—H24A	109.5
C8—C9—C12	120.9 (4)	O6—C24—H24B	109.5
C10—C9—C12	120.2 (4)	H24A—C24—H24B	109.5
C11—C10—C9	120.2 (4)	O6—C24—H24C	109.5
C11—C10—H10	119.9	H24A—C24—H24C	109.5
C9—C10—H10	119.9	H24B—C24—H24C	109.5
			
C5—N1—C2—C1	164.9 (3)	C19—C14—C15—C16	−1.2 (7)
C5—N1—C2—C3	−70.2 (4)	C13—C14—C15—C16	177.1 (4)
C4—O3—C3—O2	−2.1 (6)	C14—C15—C16—C17	−1.4 (7)
C4—O3—C3—C2	−179.7 (3)	C15—C16—C17—C18	3.1 (7)
N1—C2—C3—O2	162.9 (4)	C15—C16—C17—C20	−176.0 (4)
C1—C2—C3—O2	−71.1 (5)	C16—C17—C18—C19	−2.1 (6)
N1—C2—C3—O3	−19.4 (5)	C20—C17—C18—C19	177.0 (4)
C1—C2—C3—O3	106.5 (4)	C15—C14—C19—C18	2.2 (7)
C2—N1—C5—O1	−0.6 (5)	C13—C14—C19—C18	−176.1 (4)
C2—N1—C5—C6	−178.0 (3)	C17—C18—C19—C14	−0.6 (7)
O1—C5—C6—C7	−142.9 (4)	C22—N2—C20—O4	−3.3 (6)
N1—C5—C6—C7	34.5 (5)	C22—N2—C20—C17	178.3 (3)
O1—C5—C6—C11	35.5 (6)	C16—C17—C20—O4	−38.6 (6)
N1—C5—C6—C11	−147.1 (4)	C18—C17—C20—O4	142.3 (4)
C11—C6—C7—C8	0.2 (6)	C16—C17—C20—N2	139.8 (4)
C5—C6—C7—C8	178.5 (3)	C18—C17—C20—N2	−39.3 (5)
C6—C7—C8—C9	1.3 (6)	C20—N2—C22—C23	46.3 (5)
C7—C8—C9—C10	−1.2 (6)	C20—N2—C22—C21	−80.0 (5)
C7—C8—C9—C12	178.9 (4)	C24—O6—C23—O5	3.1 (5)
C8—C9—C10—C11	−0.3 (6)	C24—O6—C23—C22	179.1 (3)
C12—C9—C10—C11	179.6 (4)	N2—C22—C23—O5	−134.7 (4)
C9—C10—C11—C6	1.8 (6)	C21—C22—C23—O5	−7.9 (5)
C7—C6—C11—C10	−1.8 (6)	N2—C22—C23—O6	49.4 (4)
C5—C6—C11—C10	179.8 (4)	C21—C22—C23—O6	176.2 (3)

### Hydrogen-bond geometry (Å, º)

**Table:**

*D*—H···*A*	*D*—H	H···*A*	*D*···*A*	*D*—H···*A*
N1—H1···O1^i^	0.89 (1)	2.11 (2)	2.982 (4)	168 (3)
N2—H2···O4^ii^	0.89 (1)	1.93 (2)	2.799 (4)	165 (5)
C1—H1*C*···O2^i^	0.98	2.58	3.532 (6)	164
C4—H4*B*···O2^iii^	0.98	2.36	3.340 (5)	176
C21—H21*B*···O6^iii^	0.98	2.53	3.315 (5)	137
C22—H22···O5^ii^	1.00	2.38	3.380 (5)	174
C24—H24*B*···O5^iv^	0.98	2.46	3.394 (5)	158

Symmetry codes: (i) *x*+1, *y*, *z*; (ii) *x*−1, *y*, *z*; (iii) *x*, *y*, *z*−1; (iv) *x*, *y*, *z*+1.
